# A spontaneous model of spondyloarthropathies that develops bone loss and pathological bone formation: A process regulated by IL27RA^-/-^ and mutant-p53

**DOI:** 10.1371/journal.pone.0193485

**Published:** 2018-03-01

**Authors:** Denada Dibra, Xueqing Xia, Mihai Gagea, Guillermina Lozano, Shulin Li

**Affiliations:** 1 Department of Genetics, The University of Texas MD Anderson Cancer Center, Houston, TX, United States of America; 2 Department of Pediatrics, The University of Texas MD Anderson Cancer Center, Houston, TX, United States of America; 3 Department of Veterinary Medicine & Surgery, The University of Texas MD Anderson Cancer Center, Houston, TX, United States of America; Universite de Nantes, FRANCE

## Abstract

Spondyloarthropathies, the second most frequently occurring form of chronic inflammatory arthritis, affects young adults in particular. However, a proper model with which to study the biology of this disease and to develop therapeutics is lacking. One of the most accepted animal models for this disease uses HLA-B27/Hu-β2m transgenic rats; however, only 30%-50% of male HLA-B27/Hu-β2m rats develop spontaneous, clinically apparent spondylitis and have a variable time until disease onset. Here, we report a high-incidence, low-variation spontaneous mouse model that delineates how the combination of inflammatory cytokine interleukin-27 (IL-27) signaling deficiency and mitogenic signaling (mutant p53^R172H^) *in vivo*, leads to bone loss in the vertebral bodies and ossification of the cartilage in the intervertebral discs. In this human disease–like mouse model, bone loss and pathogenic bone development are seen as early as 4 months of age in the absence of inflammatory aggregates in the enthesis or intervertebral disc.

## Introduction

Spondyloarthropathies display a unique pathology in their progression from an initial inflammatory phase to an osteoproliferative/ankylosing phase. Generating an animal model that replicates pathological changes of human disease is crucial to developing therapeutics. One of the best animal models described is the rat HLA-B27 model [1]. Although this models has all three phenotypes of the disease, namely inflammatory aggregates, bone loss, and bone gain, only 30%-50% of males develop these phenotypes the time until disease onset and the severity of lesions vary among individuals [1]. IL-23 is also a culprit in this disease; others have shown that *in vivo* expression of IL-23 is sufficient to phenocopy the human disease, with development of enthesitis and entheseal new bone development [2].

While interleukin-27 (IL-27) which is in the same family as IL-23, plays a significant role in bone remodeling and arthritis, its role in axial spondyloarthropathies *in vivo* has not been investigated *in vivo* especially in the absence of arthritic triggers [3–7]. One of the functions of IL-27 is to negatively regulate IL23, RAR-related orphan receptor (ROR) γt, and Th17 differentiation [8–10]. Interestingly, both these cytokines, IL23 and IL17 have been heavily implicated in psoriatic arthritis [11–13]. Briefly, patients with active psoriatic arthritis who had inadequate response to tumor necrosis factor inhibitors, improved signs of symptoms when treated with IL17 inhibitor [12].

P53 pathway is implicated in bone and proper skeletal development. Females that are null for p53 (p53^-/-^) die during embryogenesis from failed neural tube closure leading to exencephaly and anencephaly [14]. P53 also negatively regulates osteoblast differentiation and function by repressing the expression of osterix, an osteoblast-specific transcription factor [15]. Similarly, another independent study showed that osteoblast progenitor cells with deleted mdm2 (negative regulator of p53) had elevated p53 activity, reduced proliferation, reduced levels of the master osteoblast transcriptional regulator Runx2, and reduced differentiation [16]. Mutant p53 is an oncogene that promotes tumorigenesis [17]; one mechanism by which this occurs is through mutant p53 binding to wild-type p53 and attenuating wild-type p53’s transcriptional activity (otherwise known as dominant negative functions) [18]. Furthermore, p53 negatively regulates autoimmunity via the STAT3-Th17 axis [19]. Since both the IL-27 and p53 pathway are involved in bone development and remodeling and both negatively regulate IL-17, we thought to interrogate the biological significance of the integration of IL-27 and p53 pathways via genetic means *in vivo*. We found that IL27 signaling deficiency and a mitogenic signal such as mutant p53 converge and develop bone loss in the vertebral bodies and ossification of the cartilage in the intervertebral discs, mimicking a similar phenotype seen in patients with axial spondyloarthropathies.

## Materials and methods

### Genetically-engineered mice

IL27RA^-/-^ mice (Interleukin-27 Receptor alpha) were previously donated by Dr. Frederic de Sauvage (Genentech) in a C57Bl/6 background. P53^R172H/+^ were donated from Dr. Lozano (C57Bl/6 and a small percentage of 129/SvJ background). The breeding strategy was as follows: IL27RA^-/-^ P53^R172H/+^ mice (heterozygous for p53 R172H locus) were bred to IL27RA^-/-^ P53^R172H/+^ mice and subsequently genotyped for p53 status. IL27RA^-/-^ P53^+/+^ mice with the p53 wild-type locus were simply labeled IL27RA^-/-^ throughout the study, whereas IL27RA^-/-^ P53^R172H/+^ with p53 heterozygous containing one copy of the wild-type p53 locus and one copy of the mutant knock-in p53 were labeled IL27RA^-/-^ P53^R172H/+^. Littermates were included controls. IL27RA^-/-^ P53^R172H/H^ mice were not included in the study. The cohorts were monitored over time. Genotyping was performed as described below for both p53 and IL27RA. For the cohort studies, mice were euthanized and necropsied when they reached euthanasia criteria or at the indicated time points. Research procedures of all animals were performed according with research protocols approved by the IACUC at MDACC. Mice were euthanized via CO2 followed by cervical dislocation. During imaging, mice were anesthetized with isoflurane. Mice were housed at a maximum 5 mice per cage in a pathogen-free facility, and fed standard chow diet. Both males and females were used in our studies.

### Histopathologic analysis

H&E stained tissues were examined microscopically by a pathologist at MD Anderson Cancer Center, and the severity of lesions was scored using a 0–4 grading scale as follows: grade 0 = no histologic lesion; 1 = minimal or rare lesion (or the lesion affects less than 10% of the tissue); 2 = mild or infrequent lesion (or the lesion affects 10–20% of tissue); 3 = moderate or frequent lesion (or the lesion affects 20–40% of tissue); 4 = marked, extensive or severe lesion (or the lesion affects 40–100% of tissue). Photomicrographs were taken with Nikon Eclipse Ti microscope.

### Genotyping

DNA was extracted from ear tissue, and PCR for the p53 gene was performed with use of the primer sequence as previously described by Lang et al. [18]. The presence of the p53R172 gene was confirmed by using the forward CAAGAAGAGGTCCCGTGCTG primer sequence and the reverse TTGAGCCCAGTCCACCACAT sequence. PCR primers for the absence of IL27RA were as follows: GCTTTCGTCTCCCGTGTGC (forward) and TGAGCCCAGAAAGCGAAGGA (reverse).

### Micro-CT image acquisition and analysis

CT images were acquired as previously described [20]. Briefly, these images were acquired by using five 100-ms frames at each 1° rotation increment with an x-ray tube potential and current of 80 kVp and 450 μA, respectively, via an RS-9 micro-CT scanner (GE Healthcare, London, Ontario, Canada). Two adjacent image volumes were acquired with an overlap of 40% of the field of view in the axial direction and stitched together after reconstruction. Images had a nominal isotropic resolution of 91 μm and required approximately 25 minutesof scan time. The bone was visualized with use of GEHC Microview Software. All image isosurfaces were visualized and analyzed for bone mineral density (BMD) by using the same image threshold value.

### Statistical analysis

ANOVA with Tukey as a post-hoc test was used to analyze the differences among groups. P< 0.05 was considered significant.

## Results

**Fig 1:**
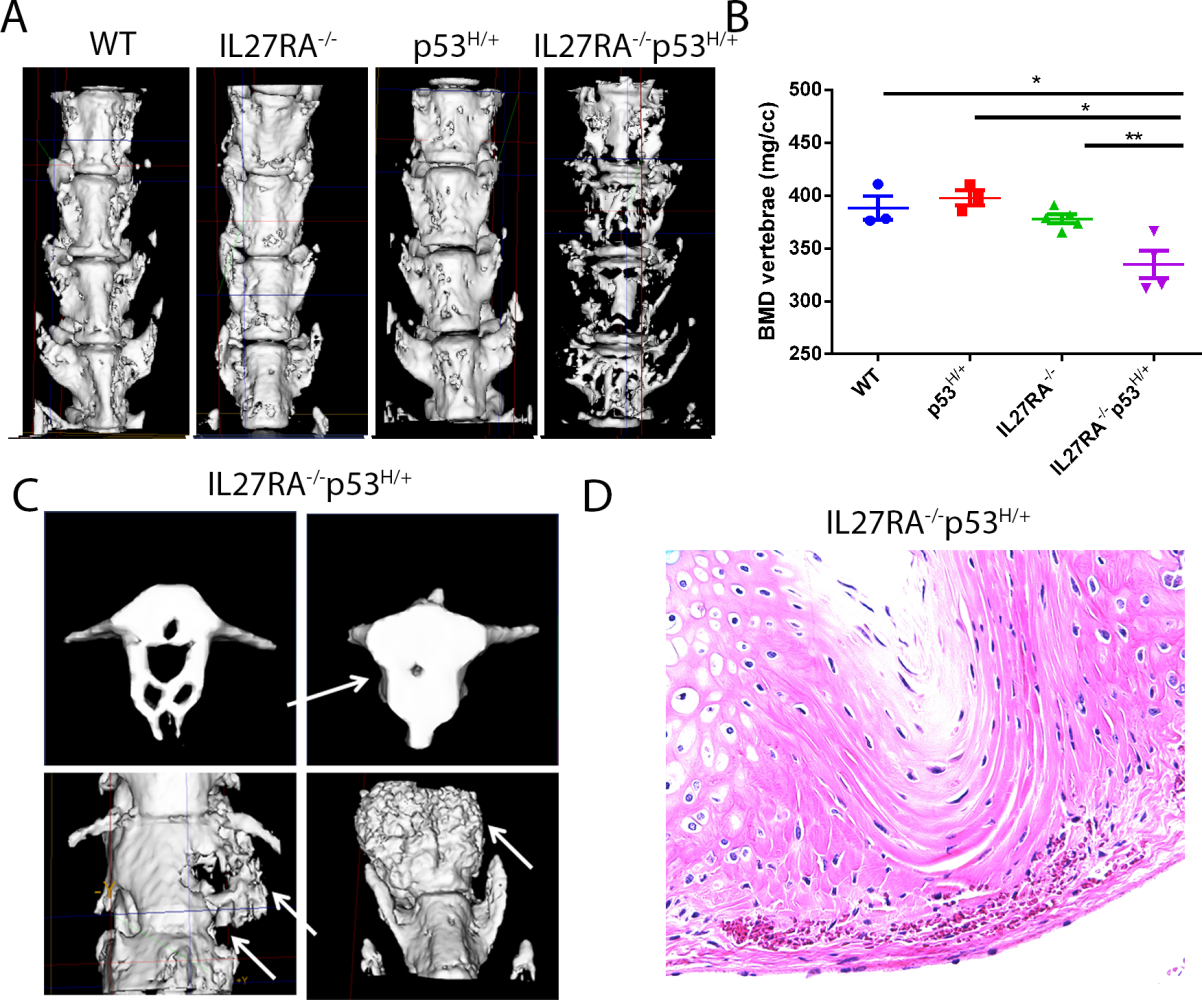
IL27RA^-/-^ p53^R172H/+^ mice spontaneously develop bone loss and pathologic bone formation when compared with controls. (A) Delineation of vertebral (V) and intervertebral disc (IVD) measurement (left). Representative micro-CT reconstruction (right) and (B) BMD measurement from the lumbar spine of aged WT, IL27RA^-/-^, p53^R172H/+^, and IL27RA^-/-^ p53^R172H/+^ mice. (C) micro-CT reconstruction portraying abnormal bone growth in the spine of aged IL27RA^-/-^ p53^R172H/+^ mice. (D) Photomicrograph of HE stained sections of the intervertebral disc of aged IL27RA^-/-^ p53^R172H/+^ mice with rare inflammatory cells within the enthesis adjacent to the intervertebral discs. *, p< 0.05. Each dot represents an individual mouse.

We bred IL27RA^-/-^ mice with mutant p53 heterozygous (p53^R172H/+^) mice to generate IL27RA^-/-^ p53^R172H/+^ mice. We micro-CT scanned aged-matched mice at 12–14 months of age. Indeed, IL27RA^-/-^ p53^R172H/+^ mice had significant loss of bones in the spinal column when compared with controls (Fig 1A and 1B). Aside from bone loss, these mice had significant bone growth as well (Fig 1C). Others have reported that mild to heavy infiltration of inflammatory aggregates between the vertebral bodies and intervertebral discs are the initiating events in the development of these bone lesions [21]. Surprisingly, infiltration of minimal to no inflammatory cells were detected in either the enthesis or in the intervertebral discs in our study, suggesting that a preliminary inflammatory event may not be required for the initiation or development of pathological lesions of the bone observed here (Fig 1D).

**Fig 2:**
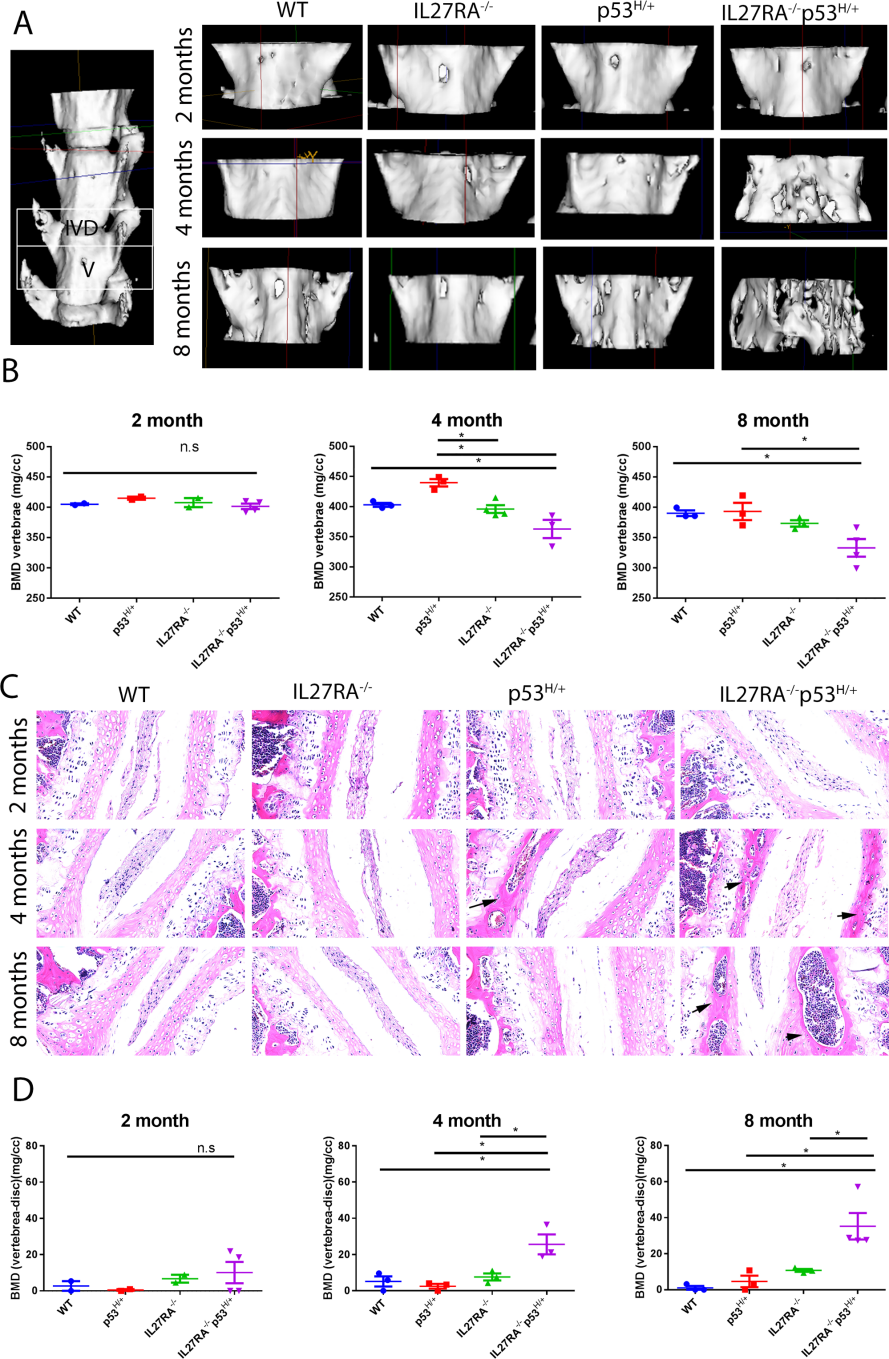
IL27RA^-/-^ p53^R172H/+^ mice spontaneously develop high incidence and low variation bone loss and ossification as early as age 4 months compared with controls. (A) Representative micro-CT reconstruction and (B) BMD measurement from the lumbar spine of 2-, 4-, and 8-month- old WT, IL27RA^-/-^, p53^R172H/+^, and IL27RA^-/-^ p53^R172H/+^ mice. (C) H&E stained images show normal intervertebral discs in WT and IL27RA^-/-^ mice, and areas of pathologic ossification of the cartilage (black arrows) of the intervertebral disc around the nucleus pulposus in IL27RA^-/-^ p53^R172H/+^ mice observed at 4 and 8 months of age. (D) BMD measurement of intervertebral discs of mice of various genotypes across time. *, p< 0.05. Each dot represents an individual mouse.

One caveat with the already well-established HLA-B27/Huβ2m rats is that these animals have low incidence of disease and variable time until disease onset [1]. To understand whether these limitations are present in the proposed IL27RA^-/-^ p53^R172H/+^ mouse model, a large number of mice were examined to delineate the incidence, locations, frequency, and variability within the group. During the life of our mice, a sequential analysis of BMD (bone mineral density) utilizing the micro-CT images was conducted. van Duivenvoorde et al. [21] showed that bone loss and bone proliferation occur in two different anatomical areas, in the vertebral body and the intervertebral space, respectively. At age 2 months, no significant changes were seen in bone loss of the spine of IL27RA^-/-^ p53^R172H/+^ mice, whereas significant bone loss was seen at age 4 and 8 months compared with the controls (Fig 2A). Both genders were included in our studies. Furthermore, size and mobility of the pups was normal across all genotypes. The observation that no differences in BMD were seen in the spinal column amongst different genotypes of mice at 2 months of age suggest that bone development was normal up to this age in IL27RA^-/-^p53^R172H/+^ mice. Therefore, most likely the lower BMD observed in IL27RA^-/-^p53^R172H/+^ is associated with bone loss rather than abnormal bone formation. To quantify these differences while uncoupling bone loss and bone growth, we set the measurement to cover only the vertebral body and to exclude the intervertebral disc. The same height for each vertebra was measured in all mice. Significant BMD decline was seen in IL27RA^-/-^ p53^R172H/+^ mice compared with the other controls starting as early as 4 months (Fig 2B). Of interest, even in the absence of IL27RA^-/-^, some degree of bone loss was observed, but it was less significant than in the IL27RA^-/-^ p53^R172H/+^ mice. Although we observed some variability within the IL27RA^-/-^ p53^R172H/+^ group, which was expected because these mice spontaneously develop these phenotypes, all of the mice tested had significant bone loss compared with controls, supporting a high incidence and reproducibility of this phenotype (Fig 2B).

Using the HLA-B27/Huβ2m rat model, van Duivenvoorde et al. [21] indicated that pathological new bone formation starts at the intervertebral discs. Therefore, cross-sagittal sections of the spinal column of mice at various ages were analyzed. No evident changes were seen in IL27RA^-/-^ p53^R172H/+^ mice or in controls at age 2 months. Of interest, ossification of the cartilage of the intervertebral disc on both sides of the nucleus pulposus of the IL27RA^-/-^ p53^R172H/+^ mice was observed in 4-month-old mice (Fig 2C). These differences were also present in 8-month-old mice. Surprisingly, even in the intervertebral disc of 4-month-old p53^R172H/+^ mice, some ossification of the cartilage in the intervertebral disc was observed, but not in the 8-month-old mice. Such observations suggest that mutant p53 might contribute to the ossification of cartilage, but the incidence of this phenotype becomes high only with the combination of mutant p53 and IL-27 signaling. To quantify these differences, BMD measurement on the intervertebral disc on microCT images of IL27RA^-/-^ p53^R172H/+^ and control mice were performed. The same height for intervertebral disc was used across all animals analyzed. BMD loss of the adjacent vertebral body was used as a baseline for the intervertebral BMD gain calculations. Indeed, BMD gain measurements associated with histological observations (Fig 2D): BMD in the intervertebral disc of IL27RA^-/-^ p53^R172H/+^ mice at ages 4 and 8 months was significantly higher than that in the control groups. Remarkably, occasional or no inflammatory cells were seen in the intervertebral discs or enthesis of these mice at any of the above-mentioned time points.

**Fig 3:**
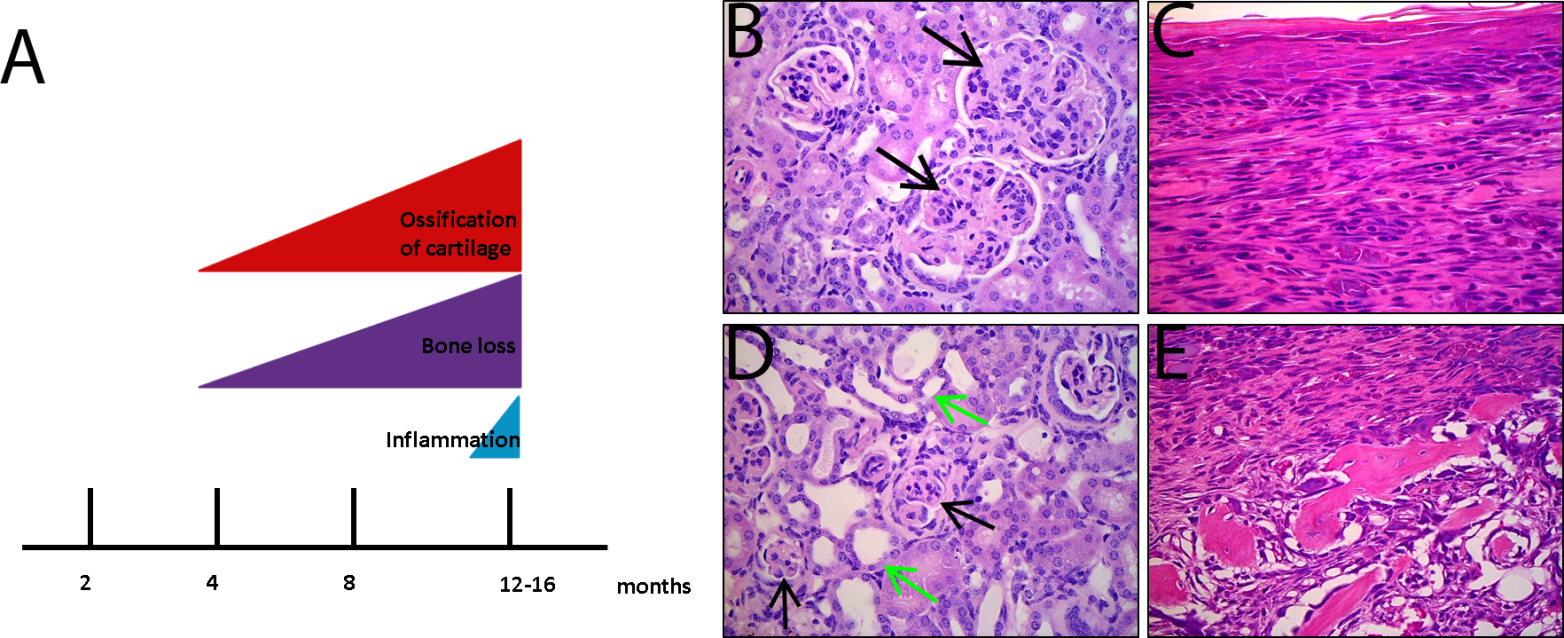
IL27RA^-/-^ p53^R172H/+^ mice develop pathologies in other organs. (A) Summary of the bone abnormalities in IL27RA^-/-^ p53^R172H/+^ mice. (B and C) Chronic nephropathy with membranoproliferative glomerulonephritis (black arrows in B), atrophic glomeruli (black arrows in C), and dilated renal tubules with atrophic epithelium (green arrows in C). (D) Skin of the tail with chronic dermatitis and fibroplasia. (E) Chronic periosteitis and osteomyelitis of the vertebral body of tail. H&E stain, magnification 400x.

Spondyloarthropathies in patients comprise a variety of related disorders including inflammatory pathologies (albeit at a low percentage) in other non-bone-related sites such as skin, lung, kidney, and intestines [22–24]. Indeed, IL27RA^-/-^ p53^R172H/+^ mice developed a few of these pathologies including chronic kidney nephropathy (7.5% incidence) (Fig 3B and 3C). Finally, 4% of IL27RA^-/-^ p53^R172H/+^ mice developed neutrophilic dermatitis in the tail that progressed into the subcutaneous tissues and vertebral body of the tail characterized by local neutrophilic periosteitis and osteitis (Fig 3D and 3E). None of these phenotypes were observed in control, age-matched mice.

## Discussion

There is extensive debate if and how inflammation and bone gain are coupled in spondyloarthropathies. Most of the described animal models support an inflammatory-driven process such as overexpression of human TNF, mouse TNF, transmembrane TNF, or overexpression of IL1, which develop a wide range of inflammation-related symptoms in multiple organs such as destructive polyarthritis, enthesitis, and gut inflammation [25–29]. Aside from the transmembrane TNF mouse model that develops some degree of new bone formation, none of the previous models have this phenotype. While IL27RA^-/-^ p53^R172H/+^ mouse model described here is different from the above-mentioned models, it also *complements* the already known mouse models as it develops primary bone loss and pathological new bone formation associated with minimal or no inflammation in these aged mice. Furthermore, it suggests that these relationships are dissociated, meaning that bone loss and pathological new bone formation can occur in the absence of inflammation. Another advantage of this model is that we can manipulate which signals contribute to bone loss (IL27RA^-/-^) or ossification of cartilage (mutant p53), therefore making it a useful tool to investigate how these two processes are interrelated or finding better drugable targets. Other strengths emerging from this model are that we can monitor in a timely fashion when each of these pathological processes starts to occur, with bone loss and ossification of the cartilage within the intervertebral disc starting as early as age 4 months and progressing with age.

Here, we present evidence *in vivo* that IL-27 could have a protective role in axial spondyloarthropathies. The protective role of IL-27 strongly supports the findings of other related chronic inflammatory models such as human RA. Briefly, IL-27 potently inhibits human osteoclastogenesis by acting on the osteoclast precursor [7]. Mechanistically, IL-27 could exert these protective effects based on the inhibition of Th17 lineage commitment [10]. Indeed, although Th17 has been closely linked to the pathogenesis of spondyloarthropathies in patients, IL-27 has been shown to exert it protective effects in a mouse model of collagen-induced arthritis [4], which is a Th17-dependent inflammatory event, but not in adjuvant or proteoglycan-induced arthritis, which are considered Th1-related events [30, 31]. And lastly, since both IL27 and p53 negatively regulate IL17 levels, we speculate that heightened levels of IL17 may be involved in our model [19].
